# Avoidance of systemic anticoagulation during intermittent haemodialysis with heparin-grafted polyacrilonitrile membrane and citrate-enriched dialysate: a retrospective cohort study

**DOI:** 10.1186/1471-2369-15-104

**Published:** 2014-07-03

**Authors:** Karlien François, Karl Martin Wissing, Rita Jacobs, Dries Boone, Kristine Jacobs, Christian Tielemans

**Affiliations:** 1 Department of Nephrology, Universitair Ziekenhuis Brussel, Vrije Universiteit Brussel (VUB), Laarbeeklaan 101, 1090 Jette, Belgium; 2 Department of Intensive Care, Universitair Ziekenhuis Brussel, Vrije Universiteit Brussel (VUB), Laarbeeklaan 101, 1090 Jette, Belgium

**Keywords:** Anticoagulation-free haemodialysis, Citrate dialysate, Heparin-grafted dialyzer, Coagulation

## Abstract

**Background:**

Since October 2010, the combination of a heparin-grafted polyacrilonitrile (AN69ST) membrane with a 0.80 mmol/L citric acid-containing dialysate is routinely used in our centre for intermittent haemodialysis, without systemic anticoagulation, in critically ill patients with increased bleeding risk. The primary outcome of this retrospective cohort study was to assess the development of circuit clotting during these dialysis procedures. Secondly, we assessed the impact of clotting on treatment duration, the incidence rate of coagulation-induced retransfusion failure and the association of patient and dialysis characteristics with the occurrence of clotting.

**Methods:**

Dialysis and patient data on consecutive intermittent haemodialysis procedures, performed at the Intensive Care Unit of Universitair Ziekenhuis Brussel between October 2010 and March 2012, were retrospectively reviewed. We used descriptive statistics as well as a random effects logit model with patient identity as a panel variable to assess associations.

**Results:**

Of a total of 309 treatments combining a heparin-grafted AN69ST membrane and a 0.8 mmol/L citric acid-enriched dialysate in 94 patients, circuit clotting was reported in 17.5% (95% CI 13.2% to 21.7%; N = 54), and in 19% (95% CI 13.6% to 24.4%; N = 40) of sessions with prescribed treatment time ≥ 4 hours (N = 210). Clotting shortened treatment time in 15.2% (95% CI 11.4% to 19.7%; N = 47) of sessions by a median of 55 (IQR 20 to 80) minutes. Complete clotting of the circuit with inability for retransfusion occurred in 4.2% (95% CI 2.2% to 7.0%; N = 13) of sessions. Circuit coagulation was not associated with APACHE II score, patient age, gender, number of treatments, type of vascular access or ultrafiltration rate.

**Conclusion:**

Intermittent haemodialysis without systemic anticoagulation combining a heparin-grafted AN69ST dialyzer with a citrate-enriched dialysate favourably compares as to clotting complications with the published outcomes of anticoagulation-free intermittent haemodialysis strategies using saline flushes, heparin-coated dialyzer in combination with regular dialysate or regional citrate anticoagulation with calcium supplemented dialysate. The incidence of circuit clotting in our cohort appears to be higher than previously reported for regional citrate anticoagulation with a calcium-free dialysate.

## Background

Haemodialysis treatment requires anticoagulation or antithrombotic treatment to prevent clotting of the extracorporeal circuit. Strategies which induce systemic anticoagulation should however be avoided in patients with bleeding risks. In this setting, the European Best Practice Guidelines recommend haemodialysis without systemic anticoagulation by using either regular saline flushing or regional citrate anticoagulation (RCA) [1].

Heparin-free haemodialysis with saline flushes requires removal of all air from dialyzer and lines during priming, prevention of air introduction in the extracorporeal system during dialysis and a high blood flow rate. After rapidly increasing extracorporeal blood flows, saline flushes are administered every 15–30 minutes into the pre-dialyzer limb. Saline boluses vary between 25 to 200 ml administered every 15 to 30 minutes. Coagulation in the circuit has been reported with a variable frequency, 5 to 50%, with early termination of the dialysis session in 5 to 7% [2-7]. Mean treatment duration varied between 3 and 4 hours [2,3,5,6]. Previous reports documented preserved clearance efficiency in haemodialysis using intermittent saline flushes [2,4,5]. The recent prospective and randomized HepZero study in maintenance dialysis patients has shown that only half of the 127 patients in the control group, receiving saline flushes of 100–300 ml every 30 minutes or predilution infusion of 1–2 litres per hour in haemodiafiltration, were able to complete 4 hours of dialysis without reaching one of the clotting-related events of the composite endpoint [7]. RCA uses the calcium inhibiting actions of trisodium citrate, infused into the arterial line of the dialyzer, to block the coagulation cascade. Calcium-citrate complexes are cleared into the dialysate and calcium-infusion in the venous return line restores plasma calcium concentration and prevents hypocalcaemia. This strategy is a safe and feasible option for intermittent haemodialysis (IHD) in children and adults with increased bleeding risk [8-11]. As an alternative approach, a citrate containing dialysate was developed by replacing some of the acetate with citrate. The use of a low-concentrated citric acid (0.8 mmol/L) dialysate does not require additional calcium supplementation, has been proven to be safe, to improve dialyzer clearance [12,13] and to decrease heparin requirements [14,15]. The use of a heparin-grafted dialyzer has also been shown to reduce heparin requirements but is less efficient in terms of clotting of the extracorporeal circuit and dialysis efficacy than RCA [16]. A recent prospective and controlled trial showed that use of the heparin-grafted polyacrilonitrile (AN69ST) dialyzer in the absence of systemic anticoagulation was complicated by circuit coagulation in 31.5% of 4-hour dialysis sessions [7]. Our hypothesis is that the combination of a heparinized dialyzer membrane with citrate-enriched dialysate might further reduce clotting risk of the extracorporeal circuit in the absence of systemic anticoagulation. This approach has not been previously investigated, but has been used routinely for IHD of patients considered at increased bleeding risk during admission at the Intensive Care Unit (ICU) of our institution. The primary endpoint of the present retrospective study was to determine the incidence rate of extracorporeal circuit clotting during haemodialysis sessions with the heparin-grafted AN69ST dialyzer in combination with a citrate-enriched dialysate.

## Methods

We performed a retrospective analysis of consecutive haemodialysis sessions without systemic anticoagulation performed in the ICU of the Universitair Ziekenhuis Brussel between October 2010 and March 2012. All IHD sessions using a heparin-grafted AN69ST dialyzer membrane (Evodial®, Gambro, Zaventem, Belgium) in combination with a 0.80 mmol/L citric acid dialysate (Citrasate®, Advanced Renal Technologies Inc., WA; distributed by Fresenius Kabi, Schelle, Belgium) performed during the time frame of the study in medical as well as surgical ICUs (12 beds each) were included for analysis. Medical records and nursing plans of all these IHD sessions were reviewed for prescribed and effective treatment duration, processed blood volume, ultrafiltration rate, type of access and observed circuit coagulation. The consulting nephrologist made assessment of bleeding risk and decision to avoid systemic anticoagulation for haemodialysis on an individual clinical basis. We were not able to systematically identify the specific reasons for the choice of heparin-free dialysis. For all patients, demographic data (age, gender) were collected and APACHE II score, calculated at every ICU admission, was used as a measure of patient’s disease severity for the dialysis sessions performed during the specific admission.

Experienced haemodialysis nurses executed the IHD using AK-200 machines (Gambro, Zaventem, Belgium) and the Evodial 1.6® dialyzer which has an effective membrane surface area of 1.65 m^2^. Priming of bloodlines and dialyzer was performed following our local protocol using 2000 ml regular saline without adding any anticoagulation. Varying formulas as regards to calcium chloride and potassium concentrations of a 1.9% citric acid concentrate were used. On the contrary to standard RCA no citrate was infused in the arterial line and no calcium was administered into the venous line during the dialysis sessions. Blood flow was adapted to the highest possible rate depending on the function of the vascular access. Dialysate flow rate was fixed per protocol at 700 ml/min. Intended duration of treatment and ultrafiltration rate varied according to medical decision.

Primary outcome was the incidence of clotting of the extracorporeal circuit defined by the forming of fibrin clots in the bubble air traps, increasing transmembrane pressure during dialysis, aspect of dialyzer and bloodlines after retransfusion or complete dialyzer clotting during the session and as reported in the nursing plans by the haemodialysis nurse. The extent of clotting to venous or arterial chamber, dialyzer membrane or whole circuit is not always reported except in case of loss of circuit. Therefore, we only recorded clotting as being present or absent without grading it except in case of inability to retransfuse the dialysis circuit’s blood to the patient. The impact of clotting on duration of dialysis treatment and the incidence rate of clotting with impossibility to retransfuse were secondary outcomes. We also evaluated association of patient and dialysis characteristics with the occurrence of circuit clotting.

Descriptive statistics were calculated as proportions for categorical variables. Continuous variables were summarized as means with standard deviation in case of normal distribution or medians with inter-quartile range (IQR) otherwise. The outcome of interest, occurrence of coagulation in the dialysis circuit, was expressed as percentage with 95% confidence interval, which is equivalent to the rate of circuit coagulation per 100 dialysis sessions. To investigate the effect of patient and treatment characteristics on circuit coagulation, data were set as a panel using the STATA xtset command with patient identity as panel variable. The effect of age, gender, treatment number, planned treatment time, ultrafiltration rate, effective blood flow and APACHE II score on circuit coagulation was assessed in random-effects logit models and results expressed as odds ratios with 95% confidence intervals using the STATA xtlogit command. All statistical analysis was realized with STATA 12 for Windows (StataCorp, College Station, TX).

The Ethical Committee of Universitair Ziekenhuis Brussel approved the study. The main objective was to retrospectively validate a treatment practice, which is not completely in line with current recommendations [1]. We did not ask written informed consent as the data were collected for retrospective validation of current practice. All data regarding the study were kept in a password-protected data file.

## Results

During the 18-month study period, a total of 316 dialysis treatments combining the heparin-grafted AN69ST membrane and the 0.80 mmol/L citrate-enriched dialysate in 96 patients were performed. Seven dialysis treatments were excluded from the analysis as the corresponding files had no information on treatment length and two also lacked information on the occurrence of clotting. Complete information was obtained for 309 sessions in 94 patients who constitute the study population. Patient characteristics are summarized in Table 1.

**Table 1:** Baseline patient characteristics

Number of patients	94
Number of treatments	309
Treatments per patient (median; IQR^1^; Range)	2 (IQR 1–4; 1–20)
Age (years)	67 ± 14
Men/Women (N;%)	59 (62.8%)/35 (37.2%)
Vascular access (N;%)	
Temporary double lumen central venous catheter	84 (89.4%)
Tunnelled double lumen central venous catheter	5 (5.3%)
AV fistula	5 (5.3%)
APACHE II score (for ICU admission)	27.1 ± 10.8

^1^IQR: Inter Quartile Range.

Dialysis was performed using a temporary double lumen central venous catheter in 84 patients (89.4%) whereas a permanent access was used in 10 patients (10.6%). Temporary venous access was used in 284 dialysis sessions and achieved through the right internal jugular vein in 27.1% (N = 77), left internal jugular vein in 7.7% (N = 22), right subclavian vein in 18.0% (N = 51), left subclavian vein in 4.9% (N = 14) or a femoral vein in 42.3% (N = 120). Median treated blood volume was 54 litres (IQR 43 to 63 litres). Effective blood flow rate was calculated for every IHD as total processed volume divided by effective treatment time. Median effective blood flow rate for all IHD was 247 ml/min (IQR 224 to 275 ml/min). Median ultrafiltration was 500 ml/h (IQR 250–625 ml/h). Mean prescribed treatment time was 221 ± 44 minutes and mean effective treatment time 210 ± 51 minutes. The scheduled treatment time was reached in 252/309 (81.6%; 95% CI 77.2% to 85.9%) of dialysis sessions and in 164/210 (78.1%; 95% CI 71.9% to 83.5%) of treatments with a prescribed length of ≥ 240 minutes.

Circuit coagulation was reported in 17.5% (N = 54; 95% CI 13.2% to 21.7%) of all sessions and in 19.0% (N = 40; 95% CI 13.6% to 24.4%) of sessions with prescribed treatment duration of 4 hours or longer (N = 210) (Table 2). This required shortening of scheduled treatment time in 47 dialysis treatments (15.2%; 95% CI 11.4% to 19.7%) by a median of 55 minutes (IQR 20 to 80 minutes). Observation of circuit coagulation happened after a median of 165 minutes (IQR 140 to 200 min). In 13 out of 54 treatments with coagulation the dialysis session had to be interrupted without possibility to retransfuse blood from the extracorporeal circuit (4.2% of all sessions; 95% CI 2.2% to 7.0%). Overall, performed treatment time was significantly lower in dialysis sessions with circuit coagulation (170 ± 49 vs. 219 ± 47 minutes; P < 0.0001) and treated blood volume was reduced to 40.5 ± 13.2 vs. 54.9 ± 13.6 litres (P < 0.0001; Table 2). Circuit coagulation, with or without possibility to retransfuse blood, was the most frequent reason for early termination of dialysis (47 out of 57 session with early termination; 82.5%). Other reasons than circuit coagulation accounted for 17.5% of premature termination of IHD, in most cases due to the need for urgent medical interventions or diagnostic procedures while the patient was on dialysis.

**Table 2:** Presence of circuit coagulation in the overall cohort

	**Absent**	**Present**	**P**
Circuit coagulation	255/309	54/309	
		17.5% (13.2 – 21.7%)^1^	
Performed treatment time (mean min ± sd)	219 ± 47	170 ± 49	< 0.0001
Treated blood volume (mean litres ± sd)	54.9 ± 13.6	40.5 ± 13.2	< 0.0001
Circuit coagulation with premature termination^2^	262/309	47/309	
		15.2% (11.4 – 19.7%)^1^	
Complete circuit coagulation^3^	296/309	13/309	
		4.2% (2.2% – 7.1%)^1^	

^1^proportion with 95% CI.

^2^shortening of scheduled dialysis session length because of circuit clotting.

^3^massive clotting with inability to retransfuse blood from extracorporeal circuit.

The occurrence of circuit coagulation was not associated with number of dialysis sessions, patient age, gender or APACHE II scores (Table 3). Circuit coagulation tended to occur more frequently in dialysis sessions with a temporary catheter as compared to a permanent access (Odds ratio 1.7; 95% CI 0.4% to 6.9%), without attaining statistical significance. There were no differences in the amount of ultrafiltration and prescribed treatment time between dialysis sessions with and without circuit coagulation (Table 3). Dialysis sessions with circuit coagulation were performed at a lower mean effective blood flow (231 ± 44 vs. 250 ± 39 ml/min; P = 0.006). Effective blood flow was also significantly lower in dialysis sessions with a temporary catheter as compared to a permanent access (244 ± 38 vs. 281 ± 45 ml/min; P < 0.0001) but remained significantly associated with circuit coagulation after adjustment for access type in the Logit regression model with a 12% reduction in the odds of circuit coagulation (P = 0.007) per 100 ml increase in effective blood flow. Unidentified patient characteristics probably contributed to circuit thrombosis. Twenty out of 54 episodes occurred in four patients. On the contrary 63 out of 96 patients had no episode of thrombosis.

**Table 3:** Association of dialysis circuit coagulation with patient and treatment characteristics

	**No circuit coagulation (N = 255)**	**Circuit coagulation (N = 54)**	**OR (95% CI)**^**1**^	**P**^**1**^
Age (years)	61.3 ± 16.0	61.3 ± 17.4	1.0 (0.98 – 1.02)	0.98
Male (N;%)	177 (69.4)	40 (74.1)	1.28 (0.58 – 2.83)	0.54
Vascular access (N;%)				
Permanent access	22 (8.6)	3 (5.6)	Ref	0.77
Temporary catheter	233 (91.4)	51 (94.4)	1.72 (0.43 – 6.92)	
Treatment number^2^	3 (1 – 6)	3 (2 – 7)	1.0 (0.94 – 1.09)	0.80
Ultrafiltration (ml/h)^2^	500 (250 – 625)	500 (333 – 625)	1.11 (0.97 – 1.28)^3^	0.13
Prescribed treatment time (min)	220 ± 46	222 ± 34	1.0 (0.99 – 1.01)	0.80
Effective blood flow (ml/min)	250 ± 39	231 ± 44	0.88 (0.80 – 0.96)^3^	0.006
APACHE II^2^	29 (22 – 37)	25 (20 – 33)	0.97 (0.94 – 1.01)	0.19

^1^Random effects logit model on panel data with patient identity as panel variable.

^2^Median with inter-quartile range.

^3^Per 100 ml increase.

## Discussion

The prevalence of acute kidney injury requiring renal replacement therapy in critically ill patients varies from 4 to 19% and is associated with a significant in-hospital mortality rate [17-20]. In patients predisposed to or demonstrating bleeding, the risk for haemorrhagic complications with the use of systemic anticoagulation for haemodialysis might be a major concern [9,21,22]. Therefore, patients with acute illnesses admitted at Intensive Care and requiring IHD might benefit from techniques avoiding systemic anticoagulation. On the other hand, patients admitted at Intensive Care requiring renal replacement therapy for acute renal failure, often present a systemic inflammatory state [17], known to be associated with activation of coagulation pathways [23].

To our best knowledge, our retrospective cohort analysis is the first report on IHD using the combination of heparin-coated dialyzer and citrate-enriched dialysate as a strategy to avoid systemic anticoagulation. Contrary to standard RCA this method does not require neither infusion of citrate into the blood lines of the extracorporeal circuit nor the administration of calcium into the venous line to neutralize the anticoagulant effect of citrate. In our method, no additional heparin is administered during the dialysis treatment. The absence of systemic effects of heparin when using a heparin-grafted dialyzer has been shown before [24]. We evaluated occurrence of circuit coagulation, duration of treatment, the impact of circuit clotting on treatment length, risk of retransfusion failure and association of coagulation with treatment and patient characteristics in 309 haemodialysis sessions performed in 94 patients admitted at ICU combining a heparin-grafted dialyzer with a citrate-enriched dialysate. This study was not designed to assess the adequacy of the IHD in terms of clearance rates. Our data show that the technique was feasible for 4 hours in 81% of sessions. Overall, various degrees of circuit coagulation occurred in 17% of sessions requiring early termination of treatment in 15%. The treatments complicated with coagulation of the circuit were significantly shorter with a lower treated blood volume and were performed at a significant lower effective blood flow rate. The first two variables are clearly consequences of coagulation whereas the latter might be considered as a consequence of thrombosis reflecting beginning thrombosis but could also be a predisposing factor for inducing or aggravating thrombotic processes. Retrospective [9] and prospective [8,16] studies have proven the safety and efficacy of RCA for IHD while reporting lesser degree of circuit coagulation compared to our retrospective data. The prospective RCT by Evenepoel et al. [16] comparing efficacy and safety of heparin-coated AN69ST membranes and RCA with (RCA3.0) and without (RCA0) a calcium supplemented dialysate reported a significant higher clotting rate necessitating premature termination of the dialysis session while using a heparin-coated AN69ST membrane compared to RCA3.0 and RCA 0 (39%, 13% and 0% respectively). These data concerned ESRD stage 5 patients with a permanent dialysis access and 42% of the cohort was prophylactically treated with low-molecular weight heparin. Our data show lesser early termination of IHD due to clotting compared to the heparin-coated AN69ST in this study for the same duration of treatment (4 hours) and a same magnitude of early termination due to clotting compared to the RCA3.0 group. Schneider et al. [8] reported a greater success rate compared to our data in acutely ill patients using a RCA with a calcium-free dialysate protocol for IHD. They reported no treatment breaks due to filter clotting but admitted minor concerns with regard to calcium, not affecting the feasibility and safety of the procedure. Retrospective analysis of the use of RCA in children [9] showed feasibility of it for median duration of 240 minutes with observation of clotting in only 1/18 treatments without need for early termination. The report focused on the beneficial effect of using RCA compared to systemic heparinization in terms of controlling bleeding or preventing bleeding complication. In our retrospective analysis, we were not able to evaluate the effect of the intervention on active bleeding or bleeding risk.

The reported rate of dialyzer clotting using the saline flush technique varies up to 50% [7,25]. Moreover, the saline-flush method has significant drawbacks such as the limitation of ultrafiltration because of fluid load and the requirement for intensive nursing supervision. The recent prospective and randomized HepZero trial [7] documented circuit clotting in more than 30% of patients receiving 4 hours of haemodialysis with a heparin-coated AN69ST in the absence of other measures to prevent clotting, suggesting that this intervention alone was insufficient for maintenance haemodialysis in the absence of systemic anticoagulation. Although the mean duration of treatment in our retrospective study population was slightly shorter compared to the mean treatment duration of the heparin grafted membrane subgroup in the HepZero trial (210 ± 51 min versus 219 ± 45 min), the lower clotting rate in our study population is probably the result of the double mode of action in reducing local thrombogenicity of the extracorporeal system. The heparin coated on the dialyzer membrane will bind antithrombin III, which in turn will block thrombin and thereby thrombin activation. The thrombin-antithrombin III complex is released into the blood stream and new antithrombin III can bind heparin. As such, thrombin activation is inhibited throughout the treatment without systemic heparinization of the patient [24]. Secondly, the local delivery of a low dose of citrate diffusing from dialysate into the blood inhibits clotting by locally chelating ionized calcium [13]. The use of a low dose citrate enriched dialysate is FDA-approved and safe: there is no significant effect of this calcium chelation on systemic ionized and total calcium [12].

Neither patient age, gender, ultrafiltration volume nor the number of treatments per patient was associated with coagulation risk in our study population. Caruana et al. [4] reported a significant higher haematocrit in patients clotting their dialyzer but concluded to a low predictive value of this parameter seen the amount of overlap of haematocrit in their cohort. These authors did not observe a significant relationship between clotting events and blood flow. We lack information on patient’s biological parameters such as haemoglobin, haematocrit, coagulation studies, antithrombin III, fibrinogen and plasma lipids, all affecting rheology of the blood flow in the extracorporeal circuit and thus being potential contributing factors in provoking circuit thrombosis. Other weaknesses of our study are the retrospective design, the absence of a (historical) control group and the selection of treatments based on individual clinical assessment rather than predefined bleeding-risk criteria. Additional limitations are variable contributions of individual patients to the overall number of dialysis sessions and the use of different types of vascular access. Besides, we did not assess dialysis efficacy in terms of clearances. Our study did not include a cost analysis of the presented technique, taking in mind higher costs for the heparin-grafted dialyzer membrane and dialysate compared to regular ones, neither an objective parameter of nursing workload or ease-of-use of this technique. Our model consists of a routine haemodialysis set-up with classical nursing supervision, without electrolyte monitoring requirements. Notwithstanding the important drawbacks of our study, we consider our data regarding a new strategy avoiding all systemic anticoagulation for intermittent haemodialysis of interest to nephrologists and other specialists in the care of patients with acute renal injury.

## Conclusions

The combination of the heparin-grafted AN69ST dialyzer with a 0.8 mmol/L citrate-enriched dialysate allowed successful haemodialysis without systemic anticoagulation for treatment times of ≥ 4 hours in 81% of patients in our study population. Despite the absence of systemic anticoagulation, only 15% of dialysis sessions were prematurely discontinued due to circuit clotting with a median reduction in treatment time of 55 minutes in sessions that had to be discontinued. Circuit coagulation made blood retransfusion impossible in only 4% of dialysis sessions. We conclude that the presented strategy favourably compares as to clotting complications with published outcomes of anticoagulation-free intermittent haemodialysis strategies using saline flushes, heparin-coated dialyzer in combination with regular dialysate or RCA with calcium supplemented dialysate. However, published data showed that RCA with a calcium-free dialysate was associated with a lower incidence of circuit coagulation than observed in our cohort, a benefit that has to be put in balance with the higher need for monitoring and nursing care of this technique. Despite the limitations of our study, we believe our data provide evidence to support future prospective controlled research on the efficacy and cost-effectiveness of the combination of a heparin-coated dialyzer with a citrate-enriched dialysate as an alternative for saline flushes and RCA in patients with increased bleeding risk. Prospective and controlled trials should also investigate whether the use of higher concentration of citric acid in the dialysate (up to 1 mmol/L instead of 0.8 mmol/L) can further increase the local anticoagulant effect at the dialyzer membrane and eventually increase the success rate of this simple and convenient protocol.

## Competing interest

The authors declare that they have no competing interests.

## Authors’ contributions

KF, KMW and CT designed the study. All authors contributed to data collection, interpretation of the data and writing of the paper. All authors approved the manuscript to be submitted to BMC Nephrology.

## Pre-publication history

The pre-publication history for this paper can be accessed here:

http://www.biomedcentral.com/1471-2369/15/104/prepub

## References

[B1] European Best Practice Guidelines Expert Group on Hemodialysis ERA Section V. Chronic intermittent haemodialysis and prevention of clotting in the extracorporal system Nephrol Dial Transplant 2002 17 Suppl 7 63 71 12386229 10.1093/ndt/17.suppl_7.63

[B2] Casati S Moia M Graziani G Cantaluppi A Citterio A Mannucci PM Ponticelli C Hemodialysis without anticoagulants: efficiency and hemostatic aspects Clin Nephrol 1984 21 2 102 105 6723110

[B3] Sanders PW Taylor H Curtis JJ Hemodialysis without anticoagulation Am J Kidney Dis 1985 5 1 32 35 10.1016/S0272-6386(85)80132-3 3881017

[B4] Caruana RJ Raja RM Bush JV Kramer MS Goldstein SJ Heparin free dialysis: comparative data and results in high risk patients Kidney Int 1987 31 6 1351 1355 10.1038/ki.1987.149 3613407

[B5] Schwab SJ Onorato JJ Sharar LR Dennis PA Hemodialysis without anticoagulation. One-year prospective trial in hospitalized patients at risk for bleeding Am J Med 1987 83 3 405 410 10.1016/0002-9343(87)90748-0 3310620

[B6] Stamatiadis DN Helioti H Mansour M Pappas M Bokos JG Stathakis CP Hemodialysis for patients bleeding or at risk for bleeding, can be simple, safe and efficient Clin Nephrol 2004 62 1 29 34 15267010 10.5414/cnp62029

[B7] Maurice L Marc D Joan F Renaud F Frederique M Nathalie L Patrick R A randomized controlled multicenter trial of a heparin-grafted polyacrylonitrile membrane for no-heparin hemodialysis versus standard-of-care: results of the HepZero Study. Late-breaking clinical trial posters ISN World Congress of Nephrology 2013 SA-PO1084 Clin J Am Soc Nephrol 2013 24 4B

[B8] Schneider M Thomas K Liefeldt L Kindgen-Milles D Peters H Neumayer HH Morgera S Efficacy and safety of intermittent hemodialysis using citrate as anticoagulant: a prospective study Clin Nephrol 2007 68 5 302 307 18044262 10.5414/cnp68302

[B9] Kreuzer M Bonzel KE Buscher R Offner G Ehrich JH Pape L Regional citrate anticoagulation is safe in intermittent high-flux haemodialysis treatment of children and adolescents with an increased risk of bleeding Nephrol Dial Transplant 2010 25 10 3337 3342 10.1093/ndt/gfq225 20466660

[B10] Apsner R Buchmayer H Gruber D Sunder-Plassmann G Citrate for long-term hemodialysis: prospective study of 1,009 consecutive high-flux treatments in 59 patients Am J Kidney Dis 2005 45 3 557 564 10.1053/j.ajkd.2004.12.002 15754278

[B11] Gubensek J Kovac J Benedik M Marn-Pernat A Knap B Ponikvar R Buturovic-Ponikvar J Long-term citrate anticoagulation in chronic hemodialysis patients Ther Apher Dial 2011 15 3 278 282 10.1111/j.1744-9987.2011.00951.x 21624076

[B12] Ahmad S Callan R Cole JJ Blagg CR Dialysate made from dry chemicals using citric acid increases dialysis dose Am J Kidney Dis 2000 35 3 493 499 10.1016/S0272-6386(00)70203-4 10692276

[B13] Kossmann RJ Gonzales A Callan R Ahmad S Increased efficiency of hemodialysis with citrate dialysate: a prospective controlled study Clin J Am Soc Nephrol 2009 4 9 1459 1464 10.2215/CJN.02590409 19661218 PMC2736688

[B14] Chanard J Lavaud S Maheut H Kazes I Vitry F Rieu P The clinical evaluation of low-dose heparin in haemodialysis: a prospective study using the heparin-coated AN69 ST membrane Nephrol Dial Transplant 2008 23 6 2003 2009 10.1093/ndt/gfm888 18156457

[B15] Sands JJ Kotanko P Segal JH Ho CH Usvat L Young A Carter M Sergeyeva O Korth L Maunsell E Zhu Y Krishnan M Diaz-Buxo JA Effects of citrate acid concentrate (citrasate(R)) on heparin N requirements and hemodialysis adequacy: a multicenter, prospective noninferiority trial Blood Purif 2012 33 1–3 199 204 22269855 10.1159/000334157

[B16] Evenepoel P Dejagere T Verhamme P Claes K Kuypers D Bammens B Vanrenterghem Y Heparin-coated polyacrylonitrile membrane versus regional citrate anticoagulation: a prospective randomized study of 2 anticoagulation strategies in patients at risk of bleeding Am J Kidney Dis 2007 49 5 642 649 10.1053/j.ajkd.2007.02.001 17472846

[B17] Uchino S Kellum JA Bellomo R Doig GS Morimatsu H Morgera S Schetz M Tan I Bouman C Macedo E Gibney N Tolwani A Ronco C Acute renal failure in critically ill patients: a multinational, multicenter study JAMA 2005 294 7 813 818 10.1001/jama.294.7.813 16106006

[B18] Hoste EA Clermont G Kersten A Venkataraman R Angus DC De Bacquer D Kellum JA RIFLE criteria for acute kidney injury are associated with hospital mortality in critically ill patients: a cohort analysis Crit Care 2006 10 3 R73 10.1186/cc4915 16696865 PMC1550961

[B19] Goldberg R Dennen P Long-term outcomes of acute kidney injury Adv Chronic Kidney Dis 2008 15 3 297 307 10.1053/j.ackd.2008.04.009 18565480

[B20] Lines SW Cherukuri A Murdoch SD Bellamy MC Lewington AJ The outcomes of critically ill patients with acute kidney injury receiving renal replacement therapy Int J Artif Organs 2011 34 1 2 9 10.5301/IJAO.2011.6312 21308666

[B21] Lazarus JM Complications in hemodialysis: an overview Kidney Int 1980 18 6 783 796 10.1038/ki.1980.197 7206461

[B22] Janssen MJ van der Meulen J The bleeding risk in chronic haemodialysis: preventive strategies in high-risk patients Neth J Med 1996 48 5 198 207 10.1016/0300-2977(96)00005-8 8710039

[B23] Franchini M Veneri D Lippi G Inflammation and hemostasis: a bidirectional interaction Clin Lab 2007 53 1–2 63 67 17323827

[B24] Chanard J Lavaud S Paris B Toure F Rieu P Renaux JL Thomas M Assessment of heparin binding to the AN69 ST hemodialysis membrane: I. Preclinical studies ASAIO J 2005 51 4 342 347 10.1097/01.mat.0000169119.06419.ed 16156296

[B25] Lohr JW Schwab SJ Minimizing hemorrhagic complications in dialysis patients J Am Soc Nephrol 1991 2 5 961 975 1760540 10.1681/ASN.V25961

